# Norcembranoidal Diterpenes from the Cultured-Type Octocoral *Sinularia numerosa*

**DOI:** 10.3390/ijms16023298

**Published:** 2015-02-03

**Authors:** Wu-Fu Chen, Chen-Ting Yin, Ching-Hsiao Cheng, Mei-Chin Lu, Lee-Shing Fang, Wei-Hsien Wang, Zhi-Hong Wen, Jih-Jung Chen, Yang-Chang Wu, Ping-Jyun Sung

**Affiliations:** 1 Department of Neurosurgery, Kaohsiung Chang Gung Memorial Hospital and Chang Gung University College of Medicine, Kaohsiung 833, Taiwan; E-Mails: ma4949@cgmh.org.tw (W.-F.C.); ma4200@cgmh.org.tw (C.-H.C.); 2 Graduate Institute of Marine Biology, National Dong Hwa University, Pingtung 944, Taiwan; E-Mails: bear790516@gmail.com (C.-T.Y.); jinx6609@nmmba.gov.tw (M.-C.L.); 3 National Museum of Marine Biology and Aquarium, Pingtung 944, Taiwan; 4 Department of Sport, Health and Leisure, Cheng Shiu University, Kaohsiung 833, Taiwan; E-Mail: lsfang@csu.edu.tw; 5 Department of Marine Biotechnology and Resources and Division of Marine Biotechnology, Asia-Pacific Ocean Research Center, National Sun Yat-sen University, Kaohsiung 804, Taiwan; E-Mails: whw@mail.nsysu.edu.tw (W.-H.W.); wzh@mail.nsysu.edu.tw (Z.-H.W.); 6 Department of Pharmacy, Tajen University, Pingtung 907, Taiwan; E-Mail: jjchen@tajen.edu.tw; 7 School of Pharmacy, College of Pharmacy, China Medical University, Taichung 404, Taiwan; 8 Chinese Medicine Research and Development Center, China Medical University Hospital, Taichung 404, Taiwan; 9 Graduate Institute of Natural Products, Kaohsiung Medical University, Kaohsiung 807, Taiwan; 10 Center for Molecular Medicine, China Medical University Hospital, Taichung 404, Taiwan

**Keywords:** sinuleptolide, cembranoidal diterpene, *Sinularia numerosa*, cytotoxicity

## Abstract

A known norcembranoidal diterpene, 5-episinuleptolide (**1**), along with a new analogue, 4α-hydroxy-5-episinuleptolide (**2**), were isolated from a cultured-type soft coral *Sinularia numerosa*. The structures of **1** and **2** were elucidated on the basis of spectroscopic methods and by comparison of the data with those of the related metabolites. Cytotoxicity of metabolites **1** and **2** against a panel of tumor cells is also described. Compound **2** exhibited moderate cytotoxicity toward CCRF-CEM cells with an IC_50_ value 4.21 μg/mL. Preliminary SAR (structure activity relationship) information was obtained from these two compounds.

## 1. Introduction

**Scheme 1:**
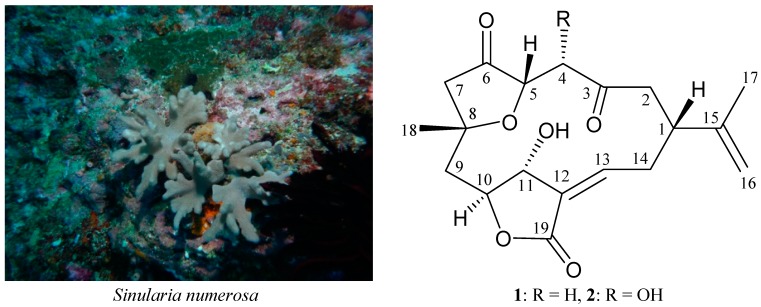
The soft coral *Sinularia numerosa* and the structures of 5-episinuleptolide (**1**) and 4α-hydroxy-5-episinuleptolide (**2**).

Previous studies on the chemical constituents of octocorals, belonging to the genus *Sinularia*, have led to the isolation of a number of interesting secondary metabolites and some of these were found to possess interesting bioactivities [1,2]. In previous studies on the chemical constituents of the soft coral *Sinularia* *numerosa* (Tixier-Durivault, 1970) (=*Sinularia crispa*) (phylum Cnidaria, class Anthozoa, order Alcyonacea, family Alcyoniidae) (Scheme 1) [3], many interesting secondary metabolites, including polyhydroxylated sterols [4,5,6], diterpenoids [6,7], sesquiterpenoids [8] and oxylipins [9] were isolated. We undertook further studies on the cultured-type *Sinularia numerosa* from which a known norcembranoidal diterpene, 5-episinuleptolide (**1**) [10,11,12,13,14,15], along with a new analogue, 4α-hydroxy-5-episinuleptolide (**2**) (Scheme 1), were isolated. In this paper, we describe the isolation, structure determination and cytotoxicity of **1** and **2**.

## 2. Results and Discussion

The known compound **1** was found to be identical with the previously reported 5-episinuleptolide (**1**) isolated from *S. leptoclados* [10,13], *S. scabra* [12], *S. lochmodes* [14], *S. maxima* [15] and *S. polydactyla* [15] by comparison of the physical (mp and ${\left[\text{α}\right]}_{\text{D}}$) and NMR data. 

**Figure 1:**
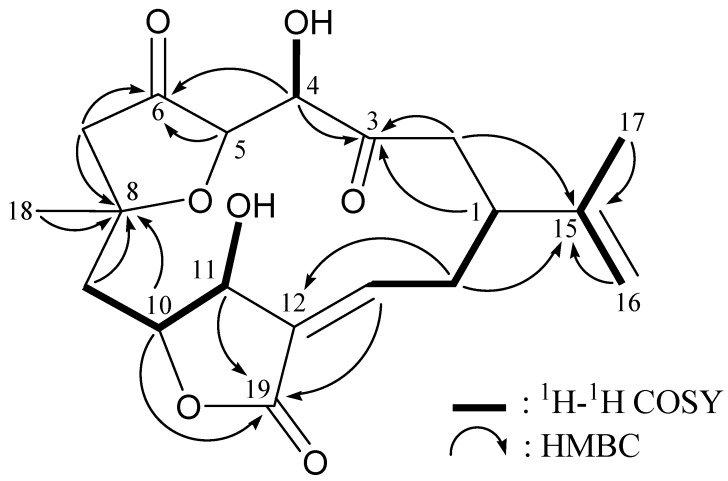
The ^1^H–^1^H COSY and key HMBC (protons→quaternary carbons) correlations for **2**.

4α-Hydroxy-5-episinuleptolide (**2**) was obtained as a white powder, ${\left[\text{α}\right]}_{\text{D}}^{20}$ − 15 (*c* 0.9, CHCl_3_). The molecular formula C_19_H_24_O_7_ was determined (8 unsaturations) based on the HRESIMS (C_19_H_24_O_7_ + Na, *m*/*z* 387.14147, calculated 387.14142). From the ^13^C NMR spectra (Table 1), **2** was found to possess an ester group (δ_C_ 169.2, C-19) and two ketone carbonyls (δ_C_ 211.5, C-6; 208.7, C-3). Two additional unsaturated functionalities were indicated by ^13^C resonances at δ_C_ 147.2 (CH-13), 147.0 (C-15), 130.9 (C-12) and 110.4 (CH_2_-16), suggesting the presence of a trisubstituted olefin and an exocyclic carbon-carbon double bond. From the ^1^H–^1^H COSY spectrum of **2** (Table 1 and Figure 1), it was possible to differentiate among the separate spin systems of H-9β/H-10/H-11, H-13/H_2_-14, H-14α/H-1 and H_2_-16/H_3_-17 (by allylic coupling). These data, together with the key HMBC correlations between protons and quaternary carbons of **2** (Table 1 and Figure 1), such as H-1, H_2_-2, H-4/C-3; H-4, H-5, H_2_-7/C-6; H_2_-7, H_2_-9, H-10, H_3_-18/C-8; H-14α/C-12; H_2_-2, H-14α, H_2_-16, H_3_-17/C-15; and H-10, H-11, H-13/C-19, permitted the elucidation of the carbon skeleton. The hydroxy proton signals at δ_H_ 3.66 (1H, d, *J* = 4.8 Hz, OH-4) and δ_H_ 2.01 (1H, br s, OH-11) were revealed by their ^1^H–^1^H COSY correlations to δ_H_ 4.34 (1H, dd, *J* = 4.8, 3.6 Hz, H-4) and 4.49 (1H, s, H-11), indicating their attachments to C-4 and C-11, respectively. Thus, compound **2** turned to be a 4-norcembranoidal diterpene possessing a γ-lactone ring, on the basis of the above analysis.

**Table 1:** ^1^H (400 MHz, CDCl_3_) and ^13^C (100 MHz, CDCl_3_) NMR data, ^1^H–^1^H COSY and HMBC correlations for norcembranoidal diterpene **2**.

Position	δ_H_ (*J* in Hz)	δ_C_, Multiple	^1^H–^1^H COSY	HMBC
1	2.80 m	44.4, CH	H-14α	C-3, -14
2	2.67–2.78 m	41.7, CH_2_	n.o. ^c^	C-1, -3, -14, -15
3		208.7, C		
4	4.34 dd (4.8, 3.6) ^a^	79.1, CH	OH-4	C-3, -6
5	4.33 br d (3.6) ^a^	76.6, CH	n.o.	C-6
6		211.5, C		
7α	2.31 d (16.8)	52.6, CH_2_	H-7β	C-6, -8, -9, -18
β	2.44 d (16.8)		H-7α	C-6, -8, -9, -18
8		80.2, C		
9α	2.10 br d (14.8)	42.5, CH_2_	H-9β	C-7, -8, -18
β	2.52 dd (14.8, 8.0)		H-9α, H-10	C-8, -10, -11, -18
10	4.67 dd (8.0, 0.8)	83.3, CH	H-9β, H-11	C-8, -11, -19
11	4.49 s	76.2, CH	H-10, OH-11	C-9, -10, -13, -19
12		130.9, C		
13	6.63 ddd (10.4, 6.8, 0.8)	147.2 CH	H_2_-14	C-11, -19
14α	2.41 ddd (13.6, 6.8, 6.8)	31.2, CH_2_	H-1, H-13, H-14β	C-1, -2, -12, -13, -15
β	3.64 ddd (13.6, 10.4, 3.6) ^b^		H-13, H-14α	C-2
15		147.0, C		
16a	4.87 d (0.8)	110.4, CH_2_	H-16b, H_3_-17	C-1, -15, -17
b	4.89 d (0.8)		H-16a, H_3_-17	C-1, -15, -17
17	1.79 s	20.8, CH_3_	H_2_-16	C-1, -15, -16
18	1.42 s	25.6, CH_3_		C-7, -8, -9
19		169.2, C		
OH-4	3.66 d (4.8) ^b^		H-4	n.o.
OH-11	2.01 br s		H-11	n.o.

^a,b^ Signals overlapping, the coupling constant for OH-4 was deduced from the coupling pattern and correlation observed between H-4 and OH-4; ^c^ n.o. = not observed.

**Figure 2:**
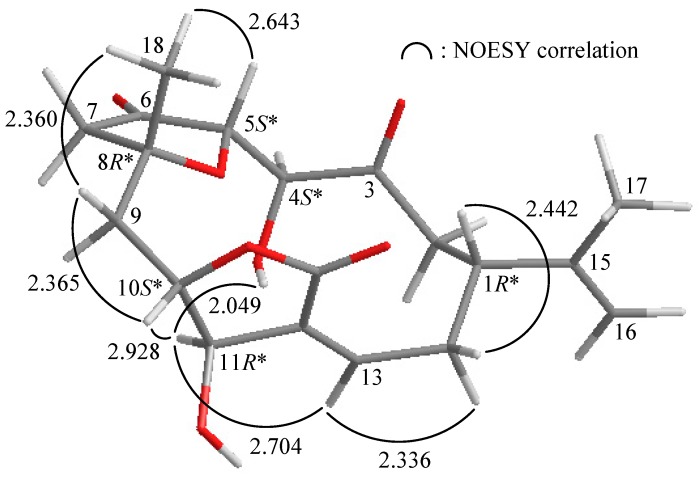
The computer-generated model of **2** using MM2 force field calculations and the calculated distances (Å) between selected protons with key NOESY correlations.

The relative configuration of **2** observed in the NOESY spectrum corroborated the MM2 force field calculations which suggested the most stable conformation as shown in Figure 2 [16]. In the NOESY experiment for **2**, it was found that one of the methylene protons at C-14 (δ_H_ 3.64) exhibited a correlation with H-1, but not with H-13, and therefore it was assigned as H-14β, and the other C-14 proton (δ_H_ 2.41) as H-14α. H-13 showed correlations with H-11 and H-14α, but not with H-1, and H-10 showed a correlation with H-11, as well as the lack of coupling was detected between H-10 and H-11, indicating the dihedral angle between H-10 and H-11 is approximately 90° and the configurations of chiral carbons C-10 and C-11 were assigned as S*- and R*-forms, respectively. One proton of C-9 methylene (δ_H_ 2.52) correlated with H-10 and H_3_-18, but not with H-11, and H_3_-18 showed a correlation with H-5, indicating that Me-18 and H-5 were β-oriented. A correlation was detected between OH-4 and H-11, indicating that the hydroxy group at C-4 should be α-oriented by modeling analysis. From the above evidences, the relative configurations of the chiral carbons of **2** were assumed to be 1R*, 4S*, 5S*, 8R*, 10S* and 11R*. 

Cytotoxicity of the norcembranoidal diterpenes **1** and **2** toward CCRF-CEM (human acute lymphoblastic leukemia), HL-60 (human acute promyelocytic leukemia), K-562 (human chronic myelogenous leukemia), U-937 (human histiocytic lymphoma), DLD-1 (human colorectal adenocarcinoma), LNCaP (human prostatic carcinoma) and MCF7 (human breast adenocarcinoma) tumor cells were studied, and the results are shown in Table 2. CCRF-CEM cell line was more sensitive to the cytotoxic effects of **2**. Compound **2** (4α-hydroxy-5-episinuleptolide) exhibited modest cytotoxicity toward CCRF-CEM cells, and was more potent than **1** (5-episinuleptolide), showing that the presence of a hydroxy substituent at C-4α position would enhance the activity.

**Table 2:** Cytotoxic data of norcembranoidal diterpenes **1** and **2**.

Compounds	Cell Lines IC_50_ (μg/mL)						
	CCRF-CEM	HL-60	K-562	U-937	DLD-1	LNCaP	MCF7
1	11.07	11.11	NA	NA	NA	NA	NA
2	4.21	10.38	18.07	10.08	NA	15.33	NA
Doxorubicin ^a^	0.01	0.01	0.35	0.05	0.49	0.10	0.16

^a^ Doxorubicin was used as a positive control and NA = not active at 20 μg/mL for 72 h.

## 3. Experimental Section 

### 3.1. General Experimental Procedures

Optical rotations were measured at a Jasco P-1010 digital polarimeter (Japan Spectroscopic Corporation, Tokyo, Japan). Infrared spectra were recorded on a Jasco FT-4100 FT-IR spectrometer (Japan Spectroscopic Corporation, Tokyo, Japan); peaks are reported in cm^–1^. NMR spectra were recorded on a Varian Mercury Plus 400 NMR spectrometer (Varian Inc., Palo Alto, CA, USA) using the residual CHCl_3_ signal (δ_H_ 7.26 ppm) as the internal standard for ^1^H NMR and CDCl_3_ (δ_C_ 77.1 ppm) for ^13^C NMR. Coupling constants (*J*) are given in Hz. ESIMS and HRESIMS were recorded using a Bruker 7 Tesla solariX FTMS system (Bruker, Bremen, Germany). Column chromatography was performed on silica gel (230–400 mesh, Merck, Darmstadt, Germany). TLC was carried out on precoated Kieselgel 60 F_254_ (0.25 mm, Merck); spots were visualized by spraying with 10% H_2_SO_4_ solution followed by heating. The normal phase HPLC (NP-HPLC) was performed using a system comprised of a Hitachi L-7110 pump (Hitachi Ltd., Tokyo, Japan) and a Rheodyne 7725 injection port (Rheodyne LLC., Rohnert Park, CA, USA). A normal phase column (Supelco Ascentis^®^ Si Cat #: 581515-U, 25 cm × 21.2 mm, 5 µm, Sigma-Aldrich. Com. St. Louis, MO, USA) was used for NP-HPLC. 

### 3.2. Animal Material

Specimens of the cultured octocoral *Sinularia* *numerosa* were collected by hand in a 0.6 ton cultivation tank with a flow-through sea water system located in the National Museum of Marine Biology and Aquarium (NMMBA), Taiwan, in 30 July 2014 and stored in freezer (−20 °C) until extraction. A voucher specimen (NMMBA-TWSC-14009) was deposited in the National Museum of Marine Biology and Aquarium, Taiwan. 

### 3.3. Extraction and Isolation

Specimens of the soft coral *Sinularia* *numerosa* (wet weight 487 g, dry weight 69 g) were minced and extracted with ethyl acetate (EtOAc). The EtOAc extract left after removal of the solvent (5.0 g) was chromatographed on silica gel by column chromatography and eluted with acetone in dichloromethane (DCM) (0%–100%, gradient) to yield 22 fractions and compound **1** (5-episinuleptolide) was obtained from fraction 9 (278.0 mg, 8:2). Fraction 13, eluted with DCM/acetone (4:6), was further purified by NP-HPLC using a mixture of DCM and acetone (5:1, flow rate: 2.0 mL/min) to yield 4α-hydroxy-5-episinuleptolide (**2**) (2.8 mg, *t*_R_ = 184 min). 

5-Episinuleptolide (**1**): colorless prisms; mp 232–233 °C ([13], 226–227 °C); ${\left[\text{α}\right]}_{\text{D}}^{22}$ −169 (*c* 0.3, CHCl_3_) (ref. [13], ${\left[\text{α}\right]}_{\text{D}}^{25}$ −119 (*c* 0.16, CHCl_3_)); IR (neat) *ν*_max_ 3265, 1748 cm^–1^; ^1^H (400 MHz, CDCl_3_) and ^13^C (100 MHz, CDCl_3_) NMR data were in full agreement with those reported previously [13].

4α-Hydroxy-5-episinuleptolide (**2**): white powder; mp 178–179 °C; ${\left[\text{α}\right]}_{\text{D}}^{20}$ −15 (*c* 0.9, CHCl_3_); IR (neat) *ν*_max_ 3421, 1740 cm^–1^; ^1^H (400 MHz, CDCl_3_) and ^13^C (100 MHz, CDCl_3_) NMR data, see Table 1, Figures S1 and S2; ESIMS: *m*/*z* 387 [M + Na]^+^; HRESIMS: *m*/*z* 387.14147 (calcd. for C_19_H_2__4_O_7_Na, 387.14142), see Figure S3–S7.

### 3.4. Molecular Mechasnics Calculations

Implementation of the MM2 force field [16] in CHEM3D PRO software from CambridgeSoft Corporation (ver. 9.0, Cambridge, MA, USA) was used to calculate molecular models.

### 3.5. MTT Antiproliferative Assay

CCRF-CEM (human acute lymphoblastic leukemia), HL-60 (human acute promyelocytic leukemia), K-562 (human chronic myelogenous leukemia), U-937 (human histiocytic lymphoma), DLD-1 (human colorectal adenocarcinoma), LNCaP (human prostatic carcinoma) and MCF7 (human breast adenocarcinoma) cells were obtained from the American Type Culture Collection (ATCC, Manassas, VA, USA). Cells were maintained in RPMI 1640 medium supplemented with 10% fetal calf serum, 2 mM glutamine and antibiotics (100 units/mL penicillin and 100 μg/mL streptomycin) at 37 °C in a humidified atmosphere of 5% CO_2_. Cells were seeded at 4 × 10^4^ per well in 96-well culture plates before treatment with different concentrations of the tested compounds. The compounds were dissolved in dimethyl sulfoxide (less than 0.02%) and made immediately of 1.25, 2.5, 5, 10 and 20 μg/μL prior to the experiments. After treatment for 72 h, the cytotoxicity of the tested compounds was determined using a MTT cell proliferation assay (thiazolyl blue tetrazolium bromide, Sigma-M2128). The MTT is reduced by the mitochondrial dehydrogenases of viable cells to a purple formazan product. The MTT-formazan product was dissolved in DMSO. Light absorbance values (OD = OD_570_ − OD_620_) were recorded at wavelengths of 570 and 620 nm using an ELISA reader (Anthos labtec Instrument, Salzburg, Austria) to calculate the concentration that caused 50% inhibition (IC_50_), *i.e.,* the cell concentration at which the light absorbance value of the experiment group was half that of the control group. These results were expressed a percentage of the control ± SD established from *n* = 4 wells per one experiment from three separate experiments [17,18,19]. 

## 4. Conclusions

Octocorals have been well recognized as an important source of potential medicinal-use agents. However, because of the octocorals are claimed to be threatened species and most of the compounds from octocorals are difficult to obtain by chemical methods at this stage, bioactive substances from cultured-type marine invertebrates will play an important role in this field. Our further studies on a cultured soft coral *Sinularia numerosa* for the extraction of additional natural substances, have led to the isolation of a new norcembranoidal diterpene, 4α-hydroxy-5-episinuleptolide (**2**), and this compound was found to exhibit modest cytotoxicity against CCRF-CEM tumor cells. This study suggested that 4α-hydroxy-5-episinuleptolide (**2**) is worthy of further biomedical investigation.

## Supplementary Materials

Supplementary materials can be found at http://www.mdpi.com/1422-0067/16/02/3298/s1.
